# Genetic Control of Resistance to *Trypanosoma brucei brucei* Infection in Mice

**DOI:** 10.1371/journal.pntd.0001173

**Published:** 2011-06-07

**Authors:** Matyáš Šíma, Helena Havelková, Lei Quan, Milena Svobodová, Taťána Jarošíková, Jarmila Vojtíšková, Alphons P. M. Stassen, Peter Demant, Marie Lipoldová

**Affiliations:** 1 Laboratory of Molecular and Cellular Immunology, Institute of Molecular Genetics, Academy of Sciences of the Czech Republic, Prague, Czech Republic; 2 Roswell Park Cancer Institute, Buffalo, New York, United States of America; 3 Faculty of Science, Charles University, Prague, Czech Republic; 4 Faculty of Biomedical Engineering, Czech Technical University in Prague, Kladno, Czech Republic; 5 Department of Genetics and Cell Biology/Clinical Genetics, Maastricht University, Maastricht, The Netherlands; Institute of Tropical Medicine, Belgium

## Abstract

**Background:**

*Trypanosoma brucei brucei* infects livestock, with severe effects in horses and dogs. Mouse strains differ greatly in susceptibility to this parasite. However, no genes controlling these differences were mapped.

**Methods:**

We studied the genetic control of survival after *T. b. brucei* infection using recombinant congenic (RC) strains, which have a high mapping power. Each RC strain of BALB/c-c-STS/A (CcS/Dem) series contains a different random subset of 12.5% genes from the parental “donor” strain STS/A and 87.5% genes from the “background” strain BALB/c. Although BALB/c and STS/A mice are similarly susceptible to *T. b. brucei*, the RC strain CcS-11 is more susceptible than either of them. We analyzed genetics of survival in *T. b. brucei-*infected F_2_ hybrids between BALB/c and CcS-11. CcS-11 strain carries STS-derived segments on eight chromosomes. They were genotyped in the F_2_ hybrid mice and their linkage with survival was tested by analysis of variance.

**Results:**

We mapped four *Tbbr* (*Trypanosoma brucei brucei* response) loci that influence survival after *T. b. brucei* infection. *Tbbr1* (chromosome 3) and *Tbbr2* (chromosome 12) have effects on survival independent of inter-genic interactions (main effects). *Tbbr3* (chromosome 7) influences survival in interaction with *Tbbr4* (chromosome 19). *Tbbr2* is located on a segment 2.15 Mb short that contains only 26 genes.

**Conclusion:**

This study presents the first identification of chromosomal loci controlling susceptibility to *T. b. brucei* infection. While mapping in F_2_ hybrids of inbred strains usually has a precision of 40–80 Mb, in RC strains we mapped *Tbbr2* to a 2.15 Mb segment containing only 26 genes, which will enable an effective search for the candidate gene. Definition of susceptibility genes will improve the understanding of pathways and genetic diversity underlying the disease and may result in new strategies to overcome the active subversion of the immune system by *T. b. brucei*.

## Author Summary

*Trypanosoma brucei* are extracellular protozoa transmitted to mammalian host by the tsetse fly. They developed several mechanisms that subvert host's immune defenses. Therefore analysis of genes affecting host's resistance to infection can reveal critical aspects of host-parasite interactions. *Trypanosoma brucei brucei* infects many animal species including livestock, with particularly severe effects in horses and dogs. Mouse strains differ greatly in susceptibility to *T. b. brucei*. However, genes controlling susceptibility to this parasite have not been mapped. We analyzed the genetic control of survival after *T. b. brucei* infection using CcS/Dem recombinant congenic (RC) strains, each of which contains a different random set of 12.5% genes of their donor parental strain STS/A on the BALB/c genetic background. The RC strain CcS-11 is even more susceptible to parasites than BALB/c or STS/A. In F_2_ hybrids between BALB/c and CcS-11 we detected and mapped four loci, *Tbbr1-4* (*Trypanosoma brucei brucei* response 1–4), that control survival after *T. b. brucei* infection. *Tbbr1* (chromosome 3) and *Tbbr2* (chromosome 12) have independent effects, *Tbbr3* (chromosome 7) and *Tbbr4* (chromosome 19) were detected by their mutual inter-genic interaction. *Tbbr2* was precision mapped to a segment of 2.15 Mb that contains 26 genes.

## Introduction

Sleeping sickness (African trypanosomiasis) continues to pose a major threat to 60 million people in 36 countries in sub-Saharan Africa. The estimated number of new cases is currently between 50 000 and 70 000 per year (WHO 2006 – African trypanosomiasis - http://www.who.int/mediacentre/factsheets/fs259/en/). The disease is caused by infection with the tsetse fly-transmitted [1] protozoan haemoflagellate *Trypanosoma brucei*, which has three major sub-species: *T. b. gambiense, T. b. rhodesiense* and *T. b. brucei.* Two of them, *T. b. gambiense* and *T. b. rhodesiense* cause sleeping sickness in humans and can also infect animals; thus domestic and wild animals are an important parasite reservoir (WHO 2006 - http://www.who.int/mediacentre/factsheets/fs259/en/). The third species, *T. b. brucei* infects a wide range of mammals, but is unable to infect humans because it lacks the SRA (serum resistance-associated) protein that prevents lysis induced by Apolipoprotein L1, which is present in normal human serum [2], [3]. *T. b. equiperdum* and *T. b. evansi,* which are derived from *T. b. brucei*, are adapted to transmission without development in tsetse fly, allowing these parasites to spread outside the African tsetse belt [4].

Upon the bite of the mammalian host by trypanosome-infected tsetse fly (*Glossina ssp*.), the parasites multiply locally in the skin and elicit a local host response in the form of an indurated skin lesion called the chancre. Eventually, the parasites enter the blood circulation via lymph vessels and can survive in the blood circulation throughout the infection of the host (reviewed in [5], [6]), remaining continually exposed to the host's immune system. *T. brucei* species have the ability to penetrate the walls of capillaries and invade interstitial tissues, but they always remain extracellular as opposed to *T. cruzi* [6]. During the meningo-encephalitic phase of the infection parasites pass into brain where they cause serious pathology [7].

African trypanosomes have evolved very sophisticated evasion mechanisms to survive in chronically infected host. These evasion mechanisms include antigenic variation of the variant surface glycoprotein (VSG) [8] and the induction of alterations in the host's defense system, such as excessive activation of the complement system leading to persistent hypocomplementemia [9], anemia, thrombocytopenia [9], down regulation of nitric oxide production [10], polyclonal B-lymphocyte activation [11], and marked immunosuppression [12], [13]. Most likely African trypanosomes induce also other, yet undiscovered, changes in the physiology of the infected host, which might interfere with effective control of the parasite [6].

Due to genetic and biological relatedness of *T. b. brucei* to other *Trypanosoma* species, many host responses to their infections are shared and therefore many aspects of human African trypanosomiasis (HAT) as well as livestock and horses infections are studied in experimental mouse infection with *T. b. brucei.* These experiments revealed great genetic variability among mouse strains in response to *T. b. brucei*, however not all results can be compared with each other because they were obtained in different experimental conditions using different *T. b. brucei* isolates. Strains DBA/2, BALB/c, BALB.B, and C3H/He are susceptible to *T. b. brucei* and display higher parasitemia, survive for a shorter time, whereas strains C57BL/10, C57BL/6, and B10.D2 are relatively resistant and survive a longer time [14], [15]. In another experiment BALB/c mice exhibited higher parasitemia than C57BL/6, but they did not differ in survival [16]. Comparison of C57BL/6 and 129/SvEv showed that 129/SvEv exhibited higher parasitemia and lower specific IgM (but not IgG) antibody levels than C57BL/6 mice. Parasitemia was higher in 129Sv/Ev, but the weight loss, mortality and the number of trypanosomes in brain was higher in C57BL/6 [7]. CBA/N mice, deficient in production of a thymus-dependent high affinity antibody subset [17] survived longer than the strains CBA/CaT6 and A/J and had slightly lower splenomegaly, but all three strains exhibited similar numbers of circulating parasites [18].

Mouse genes controlling susceptibility to trypanosomiasis caused by the subgenus *T. (Nannomonas) congolense* [19]–[22] and by sub-genus *T.* (*Schizotrypanum*) *cruzi* the causative agent of Chagas disease [23], have been successfully mapped, but a genome-wide search for susceptibility loci to the subgenus *T. (Trypanozoon) brucei* has not yet been attempted.

We have therefore analyzed the genetic control of *T. b. brucei* resistance using the recombinant congenic (RC) strains of the BALB/c-c-STS/Dem (CcS/Dem) series. This series comprises 20 homozygous strains all derived from two parental inbred strains: the “background” strain BALB/c and the “donor” strain STS. Each CcS/Dem strain contains a different, random set of approximately 12.5% genes of the donor strain STS and approximately 87.5% genes of the background strain BALB/c [24]. This series has been successfully used to study genetics of complex diseases (partly reviewed in van Wezel et al. 2001 [25]), including infection with *Leishmania major* [26]–[30] and *Bordetella pertussis* [31].

In the present work, we show that RC strain CcS-11 differs in survival from both parental strains BALB/c and STS. In the cross between BALB/c and CcS-11, we mapped four genetic loci that influence survival after *T. b. brucei* infection. Two of these loci have individual effects; the other two operate in mutual non-additive interaction. This is the first report of genetic loci controlling resistance to *T. b. brucei*.

## Materials and Methods

### Mice

Mice of strains tested for survival BALB/cHeA (BALB/c) (10 females, 10 males), STS/A (10 females, 10 males), CcS-5 (10 females, 10 males), CcS-11 (10 females, 10 males), CcS-16 (9 females, 9 males) and CcS-20 (10 females, 10 males) were 13 to 23 weeks old (mean 17, median 17) at the time of infection. Splenomegaly, hepatomegaly, body weight changes and serum levels of seven cytokines and chemokines were analyzed using females of BALB/c (22 infected, 22 non-infected), STS (17 infected, 13 non-infected) and CcS-11 (25 infected, 26 non-infected), which were 8 to 19 week old (mean 13, median 13) at the time of infection. When used for these experiments, CcS/Dem strains passed more then 38 generation of inbreeding and therefore were highly homozygous. The regions of RCS' genomes inherited from the BALB/c or STS parents were defined [32]. 169 F_2_ hybrids between CcS-11 and BALB/c (age 22 and 23 weeks at the time of infection) were produced at the Institute of Molecular Genetics. They comprised 85 females and 84 males and were tested simultaneously as a single experimental group. During the experiment, mice were placed into individually ventilated cages behind a barrier. The research had complied with all relevant European Union guidelines for work with animals and was approved by the Institutional Animal Care Committee of the Institute of Molecular Genetics AS CR and by Departmental Expert Committee for the Approval of Projects of Experiments on Animals of the Academy of Sciences of the Czech Republic.

### Parasites

The strain of *Trypanosoma brucei brucei* (AnTar1) was a generous gift of Jan van den Abbeele, Institute of Tropical Medicine “Prince Leopold”, Antwerp, Belgium. Parasites stored in liquid nitrogen were thawed and used to infect BALB/c males by intraperitoneal inoculation. Four to five days after infection, 10 µl of tail blood was collected, diluted in 90 μl of 1% formaldehyde in PBS, and the trypanosomes were counted in a Bürker counting chamber. Subsequently, tail blood was diluted in RPMI containing L-glutamine, sodium bicarbonate and glucose (Cat. Nr. R8758, Sigma, St. Louis, MO) in order to contain appropriate numbers of parasites for inoculation (Please see below).

### Trypanosomiasis challenge

Mice were inoculated intraperitoneally with 2.5×10^4^ bloodstream forms of *T. b. brucei* (AnTar1 strain) in 50. µl of RPMI containing L-glutamine, sodium bicarbonate and glucose (Cat. Nr. R8758, Sigma, St. Louis, MO). Survival time was measured in days following the day of challenge (day 0).

### Disease phenotype

In the mice infected with *T. b. brucei*, 90 µl of blood were obtained 2 days after infection for determination of cytokine and chemokine levels. Mice were killed 10 days after inoculation. The blood, spleen, and liver were collected for the further analysis.

### Cytokine and chemokine levels

Levels of GM-CSF (granulocyte-macrophage colony-stimulating factor), CCL2 (chemokine (C-C motif) ligand 2)/MCP-1 (monocyte chemotactic protein-1), CCL3/MIP-1α (macrophage inflammatory protein-1α), CCL4/MIP-1β (macrophage inflammatory protein 1-β), CCL5/RANTES (regulated upon activation, normal T-cell expressed, and secreted), CCL7/MCP-3 (monocyte chemotactic protein-3) and TNF-α, in serum were determined using Mouse chemokine 6-plex kit (Bender MedSystems, Vienna, Austria) and Mouse TNF-α simplex kit as multiplex assay. The kit contains two sets of beads of different size internally dyed with different intensities of fluorescent dye. The set of small beads is used for GM-CSF, CCL5/RANTES, CCL4/MIP-1β and TNF-α and set of large beads for CCL3/MIP-1α, CCL2/MCP-1 and CCL7/MCP-3. The beads are coated with antibodies specifically reacting with each of the analytes (chemokines) to be detected in the multiplex system. A biotin secondary antibody mixture binds to the analytes captured by the first antibody. Streptavidin – Phycoerythrin binds to the biotin conjugate and emits fluorescent signal. Test procedure was performed in the 96 well filter plates (Millipore, Billerica, MA, USA) according to the protocol of Bender MedSystem. Beads were analyzed on flow cytometer LSR II (BD Biosciences, San Jose, CA, USA). Concentrations of cytokines were determined by Flow Cytomix Pro 2.4 software. The limit of detection of each analyte was determined to be for GM-CSF 12.2 pg/ml, CCL2/MCP-1 42 pg/ml, CCL7/MCP-3 1.4 pg/ml, CCL3/MIP-1α 1.8 pg/ml, CCL4/MIP-1β 14.9 pg/ml, CCL5/RANTES 6.1 pg/ml, TNF-α2.1 pg/ml respectively.

### Genotyping of F_2_ mice

DNA was isolated from tails using a standard proteinase procedure. The strain CcS-11 differs from BALB/c at STS-derived regions on eight chromosomes [32]. These differential regions were typed in the F_2_ hybrid mice between CcS-11 and BALB/c using 14 microsatellite markers (Research Genetics, Huntsville, AL, and Generi Biotech, Hradec Králové, Czech Republic): D1Mit403, D3Mit45, D7Mit25, D7Mit18, D7Mit282, D7Mit259, D8Mit85, D10Mit46, D10Mit12, D12Mit37, D16Mit73, D19Mit51, D19Mit60 and D19Mit46 (Table S1). The average distance between any two markers in the chromosomal segments derived from the strain STS or from the nearest BALB/c derived markers was 8.7 cM. DNA was amplified in a 20-µl PCR reaction with 0.11 µM of forward and reverse primer, 0.2 mM concentration of each dNTP, 1.5 mM MgCl_2_ (except marker D7Mit259, for which the optimal concentration was 2.5 mM), 50 mM KCl, 10 mM Tris-HCl (pH 8.3), and 0.5 U of Perfect Taq Red Polymerase (Central European Biosystems, Prague, Czech Republic) and approximately 40 ng of tail DNA. PCR reaction was performed using the DNA Engine Dyad® Peltier Thermal Cycler (Bio-Rad, Hercules, CA), according to the following scheme: an initial hot start 3 min at 94°C, followed by 40 cycles of 94°C for 30 s for denaturing, 55°C for 60 s for annealing (except marker D7Mit259, for which optimal Ta = 52°C), 72°C for 60 s for elongation, and finally 3 min at 72°C for final extension. Each PCR product was electrophoresed in 3% agarose gel containing 80% of MetaPhor® Agarose (Cambrex Bio Science Rockland, Inc., Rockland, ME) and 20% of UltraPure™ Agarose (Invitrogen, Carlsbad, CA) for 15 min to 2 h at 150 V.

### Precision mapping of *Tbbr2*

To map precisely *Tbbr2* on STS derived segment of strain CcS-11 on proximal part of chromosome 12 [32] we used 8 microsatellite markers: D12Mit10a, D12Mit11, D12Mit209, D12Mit182, D12Mit104, D12Mit240, D12Mit170, *Dtnb* (dystrobrevin, beta) and 4 SNPs; rs48212577, rs4229232, rs50154157 and rs50776991 (Generi Biotech, Hradec Králové, Czech Republic). The conditions of PCR reaction were as described in the section Genotyping of F_2_ mice.

Polymorphism of SNPs was tested by restriction analysis after PCR reaction using following restriction enzymes (New England BioLabs, Ipswich, MA): HpyAV for rs48212577 (14,13 µl of PCR product, 2 U (1 µl) of HpyAV, 1.7 µl of 10x NEB buffer 4 [200 mM Tris-acetate, 500 mM Potassium Acetate, 100 mM Magnesium Acetate, 10 mM Dithiothreitol, pH 7.9], 0.17 µl of 10 mg/ml BSA (bovine serum albumin), 37°C, o/n); HinfI for rs4229232 (14.8 µl of PCR product, 5 U (0,5 µl) of HinfI, 1.7 µl of 10x NEB buffer 4, 37°C, o/n); BsmFI for rs50154157 (14,13 µl of PCR product, 2 U (1 µl) of BsmFI, 1.7 µl of 10x NEB buffer 4, 0.17 µl of 10 mg/ml BSA, 65°C, o/n), and Tsp509I for rs50776991 (14,8 µl of PCR product, 2 U (0,5 µl) of Tsp509I, 1.7 µl of 10x NEB buffer 1 [100 mM Bis-Tris-propane-HCl, 100 mM MgCl_2,_ 10 mM Dithiothreitol, pH 7.0], 65°C, o/n). The products were electrophoresed in 3% agarose gel containing 80% of MetaPhor® Agarose (Cambrex Bio Science Rockland, Inc., Rockland, ME) and 20% of UltraPure™ Agarose (Invitrogen, Carlsbad, CA) for 15 min to 2 h at 150 V.

### Statistical analysis

For the strain pattern analyses, differences in survival after *T. b. brucei* infection were compared between the RC strains CcS-5, CcS-11, CcS-16 and CcS-20 and the parental strains BALB/c and STS by Kaplan-Meier estimator using the PROC LIFETEST procedure of the SAS 9.1 statistical package for Windows (SAS Institute, Inc., Cary, NC). The differences between strains BALB/c, STS and CcS-11 in splenomegaly, hepatomegaly and body weight change were evaluated by the analysis of variance (ANOVA) and Newman-Keuls multiple comparison using the program Statistica for Windows 8.0 (StatSoft, Inc., USA). Strain and age were fixed factors and individual experiments were considered as a random parameter. The differences in parameters between strains were evaluated using the Newman-Keuls multiple comparison test at 95% significance. Differences between strains BALB/c, STS and CcS-11 in chemokine and cytokine levels were calculated by Mann Whitney U test.

Linkage of microsatellite markers with survival after *T. b. brucei* infection in F_2_ hybrids was examined by analysis of variance (ANOVA, PROC GLM statement of the SAS 8.2 for Windows (SAS Institute, Inc., Cary, NC)). Log_10_ transformation was performed in order to obtain normal distribution. The effect of each marker, sex and experiment on mouse survival was tested. Each individual marker and its interactions with other markers and sex or experiment were subjected to ANOVA. A backward elimination procedure [33] was used. The first round of the backward elimination procedure results in a list of significant markers and a list of interactions. This list (the markers and interactions with *P* value smaller than 0.05) is the input for the second round of ANOVA and the marker (or interaction) bearing the highest *P* value (if *P*>0.05) is eliminated. The backward elimination procedure was repeated till the final set of significant markers and interactions was obtained.

To obtain genome-wide significance values (corrected *P*), the observed *P*-values (α_T_) were adjusted according to Lander and Schork [34] using the formula:
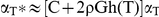



where G = 1.75 Morgan (the length of the segregating part of the genome: 12.5% of 14 M); C = 8 (number of chromosomes segregating in cross between CcS-11 and BALB/c, respectively); ρ = 1.5 for F_2_ hybrids; h(T)  = the observed statistic (F ratio).

## Results

### Differences among mouse strains in survival after *T. b. brucei* infection

**Figure 1:**
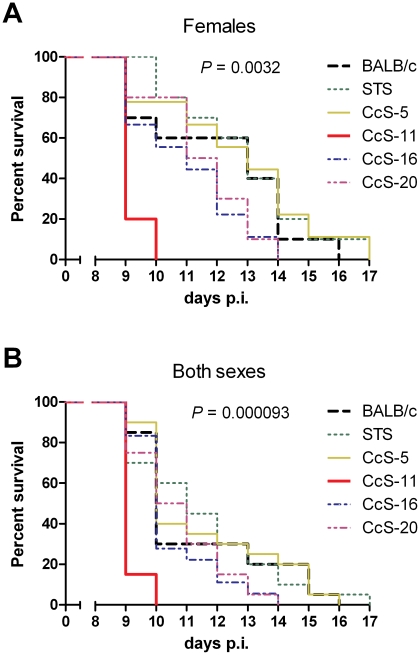
Differential survival of BALB/c, STS and selected RC strains after *T. b. brucei* infection. Survival of A. females or B. both sexes after intra-peritoneal inoculation of 2.5×10^4^ bloodstream forms of *T. b. brucei*. 10 females and 10 males from each strain were used for experiment. The only exception was strain CcS-16, where we infected 9 females and 9 males.

We have compared survival of strains BALB/c, STS/A, CcS-5, CcS-11, CcS-16 and CcS-20 after infection with *T. b. brucei*. Parental strains BALB/c and STS did not differ in survival. RC strains CcS-5, CcS-16, and CcS-20 did not significantly differ in survival from the background parental strain BALB/c. CcS-11 mice exhibit shorter survival than BALB/c mice after challenge with *T. b. brucei* infection (*P* = 0.0032 females, *P* = 0.000093 both sexes) (Figure 1 A,B). Some BALB/c mice survived up to 16 days, whereas none of the CcS-11 mice lived longer than 10 days. Strain CcS-11 was therefore selected for further analysis.

**Figure 2:**
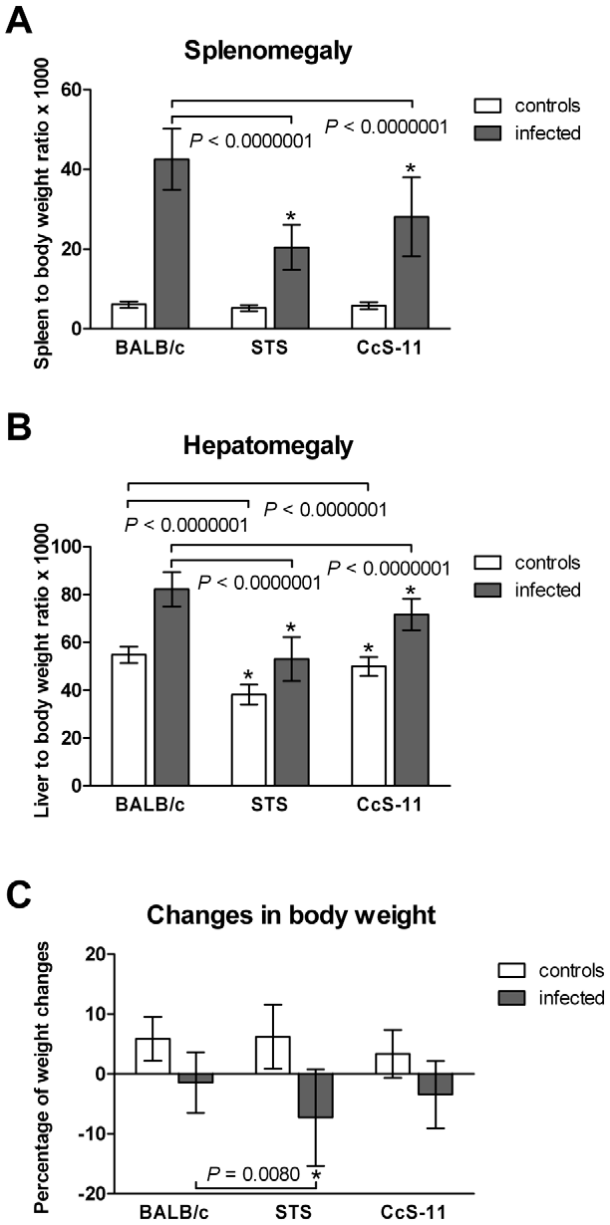
Differences in splenomegaly, hepatomegaly and changes in body weight after *T. b. brucei* infection. Female mice strains of BALB/c (17 infected, 16 non-infected), STS (17 infected, 13 non-infected) and CcS-11 (18 infected, 16 non-infected) were compared. Animals were intra-peritoneally inoculated with 2.5×10^4^ bloodstream forms of *T. b brucei.* Control, non-infected mice were kept in the same animal facility. Both groups were killed after 10 days of infection. The data show the means ± SD from three independent experiments. Asterisks indicate significant difference from BALB/c.

**Figure 3:**
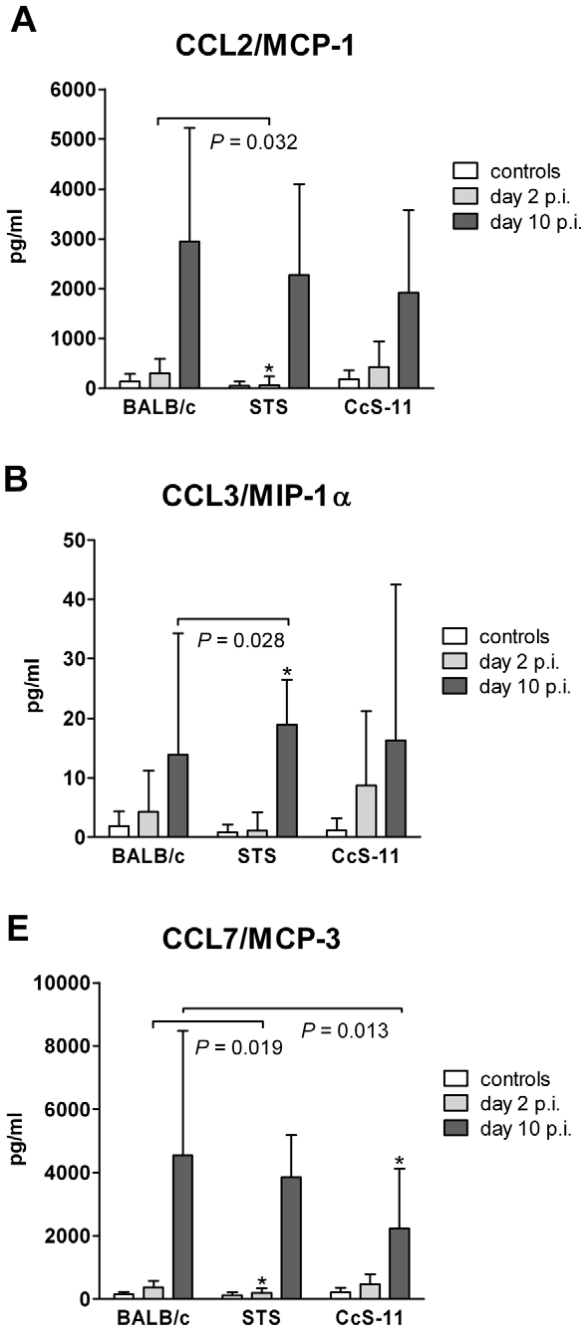
Differences in chemokine levels in strains BALB/c, STS and CcS-11 after *T. b. brucei* infection. Female mice strains of BALB/c (11 infected tested 2^nd^ day p.i., 22 infected tested 10^th^ day p.i., 22 non-infected), STS (9 infected tested 2^nd^ day p.i., 17 infected tested 10^th^ day, 13 non-infected) and CcS-11 (14 infected tested 2^nd^ day p.i., 25 infected tested 10^th^ day p.i., 26 non-infected) were compared. Animals were intra-peritoneally inoculated with 2.5×10^4^ bloodstream forms of *T. b. brucei.* Control, non-infected mice were kept in the same animal facility. Mice were killed 10 days after inoculation. The data show the means ± SD from three independent experiments. Asterisks indicate significant difference from BALB/c.

We have compared splenomegaly, hepatomegaly, changes of body weight (Figure 2), and differences in cytokine and chemokine levels (Figure 3) in females of background strain BALB/c, donor strain STS and RC strain CcS-11. Non-infected mice do not differ in spleen to body weight ratio (Figure 2A) and in changes of body weight (Figure 2C), whereas liver to body weight was higher in BALB/c than in both STS (*P*<0.0000001) and CcS-11 (*P*<0.0000001) (Figure 2B). Infection led to a significant enlargement of spleens (BALB/c: *P* = 0.000001; STS: *P* = 0.000004; CcS-11: *P* = 0.000001) and livers (BALB/c: *P* = 0.000001; STS: *P* = 0.0007; CcS-11: *P* = 0.000001) in all tested strains and to decrease of body weight (BALB/c: *P* = 0.00068; STS: *P* = 0.000044; CcS-11: *P* = 0.00037) in comparison with non-infected mice. BALB/c exhibited higher splenomegaly than STS (*P*<0.0000001) and CcS-11 (*P*<0.0000001) and also higher hepatomegaly than both STS (*P*<0.0000001) and CcS-11 (*P*<0.0000001). Differences in changes in body weight during the infection were observed between BALB/c and STS (*P* = 0.0080).

Serum levels of CCL7/MCP-3, CCL2/MCP-1, CCL3/MIP-1α, CCL4/MIP-1β, CCL5/RANTES, GM-CSF and TNF-α were measured at day 2 and 10 p.i. and compared with cytokines and chemokines serum levels of non-infected control mice. We did not observe any differences in GM-CSF levels between infected and non-infected mice. At day 2 p.i. all tested strains had increased levels of CCL7/MCP-3 in comparison with controls and in STS was also observed increased level of CCL5/RANTES. At day 10 p.i. all three tested strains exhibited increase of CCL7/MCP-3, CCL2/MCP-1, CCL3/MIP-1α, CCL4/MIP-1β, CCL5/RANTES, and TNF-α (Table S2, Figure 3, Figure S1). In infected mice, strain differences from BALB/c were observed in serum levels of CCL2/MCP-1, CCL3/MIP-1α and CCL7/MCP-3 (Figure 3). STS mice had lower serum level of CCL2/MCP-1 day 2 p.i. (*P* = 0.032) (Figure 3A) and higher level of CCL3/MIP-1α day 10 p.i. (*P* = 0.028) (Figure 3B) than BALB/c. STS mice had lower serum level of CCL7/MCP-3 than BALB/c day 2 p.i. (*P* = 0.019), whereas CcS-11 had lower serum level of this chemokine than the background parental strain BALB/c day 10 p.i. (*P* = 0.013) (Figure 3C).

### Genetic loci that control survival after infection with *T. b. brucei*

We examined survival after *T. b. brucei* infection in 169 F_2_ hybrids between the strains BALB/c and CcS-11. The strain CcS-11 differs from BALB/c in the genetic material at 8 chromosomes that were received from STS [32]. These differential STS-derived segments were genotyped in the F_2_ hybrid mice using 14 microsatellite markers. Statistical analysis of linkage revealed four genetic loci that influence survival after *T. b. brucei* infection. Two of these loci have individual effects (Table 1); the other two operate in mutual non-additive interaction (Table 2). The effects of all loci were more expressed in females than in males.

**Table 1:** Loci controlling survival after *T. b. brucei* infection - Single gene effect.

Marker	Group	Genotype						*P* value	corr. *P* value
		CC		CS		SS			
**D3Mit45**	females	15.88±0.36	(n = 18)	16.98±0.41	(n = 48)	19.58±0.93	(n = 19)	0.0010	**0.0494**
**(** ***Tbbr1*** **)**	both sexes	15.17±0.35	(n = 39)	15.73±0.33	(n = 96)	17.22±0.40	(n = 34)	0.0077	NS (0.267)
D8Mit85	females	18.32±0.86	(n = 20)	15.99±0.38	(n = 44)	18.03±0.85	(n = 20)	0.0011	0.0542 (suggestive)
	both sexes	16.98±0.40	(n = 43)	15.14±0.35	(n = 89)	15.99±0.37	(n = 36)	0.0024	NS (0.0994)
**D12Mit37**	females	19.23±0.90	(n = 15)	17.29±0.43	(n = 53)	15.89±0.36	(n = 15)	0.0004	**0.0224**
**(** ***Tbbr2*** **)**	both sexes	17.06±0.40	(n = 32)	15.74±0.27	(n = 98)	15.38±0.35	(n = 37)	0.0013	0.0583 (suggestive)

F_2_ hybrids between CcS-11 and BALB/c were tested. Means and standard errors of means of survival times and *P*-values were computed by analysis of variance. Logarithmic (Log_10_) transformation was used to obtain normal distribution and the obtained values were retransformed after calculation. Number of tested mice is shown in brackets.

**Table 2:** Interaction between loci that control survival after *T. b. brucei* infection.

**A. Females**						
			*P* = 0.0009		**Corrected ** ***P*** ** = 0.0332**	
		**D19Mit51 (** ***Tbbr4*** **)**				
		**CC**		**CS**		**SS**
**D7Mit282**	**CC**	18.46±1.32		18.79±0.88		16.08±0.74
**(** ***Tbrr3*** **)**		(n = 6)		(n = 11)		(n = 9)
	**CS**	18.14±0.85		17.77±0.86		17.01±0.81
		(n = 9)		(n = 18)		(n = 9)
	**SS**	14.90±1.06		17.33±0.82		18.66±0.88
		(n = 5)		(n = 9)		(n = 6)
			*P* = 0.0016		**Corrected ** ***P*** ** = 0.0555**	
		**D19Mit60**				
		**CC**		**CS**		**SS**
**D8Mit85**	**CC**	18.96±1.36		16.68±0.78		19.45±1.39
		(n = 5)		(n = 8)		(n = 7)
	**CS**	17.06±0.76		15.05±0.36		15.94±0.77
		(n = 10)		(n = 23)		(n = 11)
	**SS**	16.96±1.20		20.13±1.44		17.16±0.78
		(n = 5)		(n = 7)		(n = 8)
**B. Both sexes**						
			*P* = 0.0013		**Corrected ** ***P*** ** = 0.0430**	
		**D19Mit51 (** ***Tbbr4*** **)**				
		**CC**		**CS**		**SS**
**D7Mit282**	**CC**	16.63±0.79		17.02±0.40		15.24±0.72
**(** ***Tbbr3*** **)**		(n = 11)		(n = 19)		(n = 12)
	**CS**	16.44±0.79		16.21±0.39		15.70±0.37
		(n = 19)		(n = 40)		(n = 24)
	**SS**	13.99±0.67		16.48±0.38		16.90±0.80
		(n = 9)		(n = 22)		(n = 10)
			*P* = 0.0420		**Corrected ** ***P*** ** = NS**	
		**D19Mit60**				
		**CC**		**CS**		**SS**
**D8Mit85**	**CC**	18.20±0.85		16.11±0.75		16.90±0.80
		(n = 8)		(n = 18)		(n = 17)
	**CS**	15.92±0.31		14.59±0.33		14.96±0.35
		(n = 20)		(n = 49)		(n = 20)
	**SS**	15.84±0.76		16.90±0.80		15.24±0.72
		(n = 9)		(n = 16)		(n = 11)

F_2_ hybrids between CcS-11 and BALB/c were tested. Means and standard errors of means of survival times and *P*-values were computed by analysis of variance. Logarithmic (Log_10_) transformation was used to obtain normal distribution and the obtained values were retransformed after calculation. Number of tested mice is shown in brackets.

**Figure 4:**
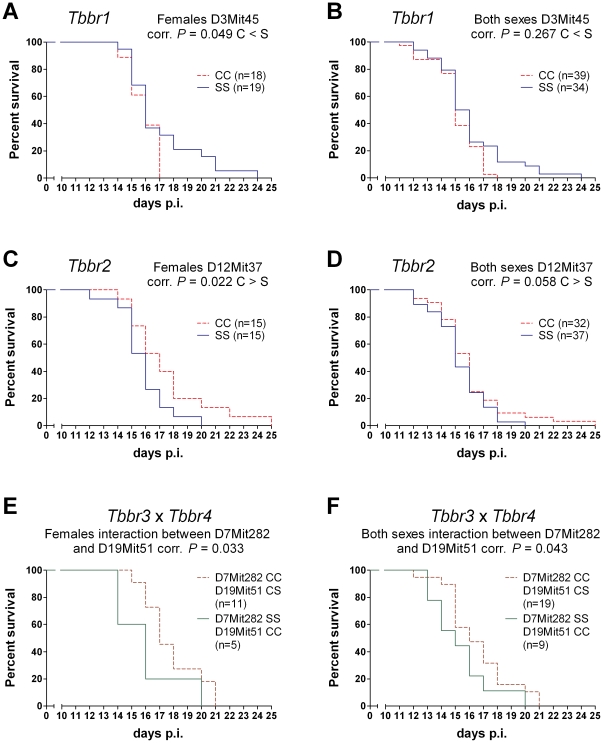
Differential survival of F_2_ hybrid mice after *T. b. brucei* infection. Mice were intra-peritoneally inoculated by 2.5×10^4^ bloodstream forms of *T. b. brucei*. **A.** females and **B.** both sexes carrying BALB/c or STS homozygous alleles in *Tbbr1* (D3Mit45); **C.** females and **D.** both sexes carrying BALB/c or STS homozygous alleles in *Tbbr2* (D12Mit37); **E.** females and **F.** both sexes carrying interacting STS homozygygous alleles in *Tbbr3* (D7Mit282) and BALB/c homozygous alleles in *Tbbr4* (D19Mit51) or BALB/c homozygous alleles in *Tbbr3* and heterozygotes in *Tbbr4.* n, number of mice.

Two loci, *Tbbr1* (*Trypanosoma brucei brucei* response 1) linked to D3Mit45 (corrected *P* value = 0.0494 females; corr. *P* = 0.267 both sexes) and *Tbbr2* linked to D12Mit37 (corrected *P* value = 0.0224 females; corr. *P* value = 0.0583 both sexes) have main effects on survival that are not dependent on or influenced by interaction with other genes (main effects) (Table 1, Figure 4 A,B,C,D). These loci have in CcS-11 an opposite effect on the studied trait. The homozygosity for the STS allele of *Tbbr1* (SS) determines about 4 days longer survival than homozygosity of the BALB/c allele (CC), whereas homozygosity for the STS allele of *Tbbr2* (SS) is associated with about three days shorter survival than the homozygosity of the BALB/c allele (CC). We have also observed a suggestive linkage of survival to D8Mit85 (corrected *P* value = 0.0542 females; corr. *P* = 0.0994 both sexes), heterozygotes had the shorter survival (Table 1).

*Tbbr3* linked to D7Mit282 influences survival in interaction with *Tbbr4* linked to D19Mit51 (corrected *P* = 0.0332 females; corr. *P* = 0.0430 both sexes). F_2_ mice with homozygous BALB/c (CC) alleles at *Tbbr3* and STS (SS) alleles at *Tbbr4* or homozygous for STS allele at *Tbbr3* and homozygous for BALB/c alleles in *Tbbr4* have the shorter survival in comparison with other combinations of *Tbbr3* and *Tbbr4* STS and BALB/c alleles (Table 2, Figure 4 E,F). A suggestive linkage was detected in females in interaction of D8Mit85 and D19Mit60 (corrected *P* = 0.0555), shorter survival has been observed in mice heterozygous both in D8Mit85 and D19Mit60 (Table 2).

### Precision mapping of *Tbbr2*

**Figure 5:**
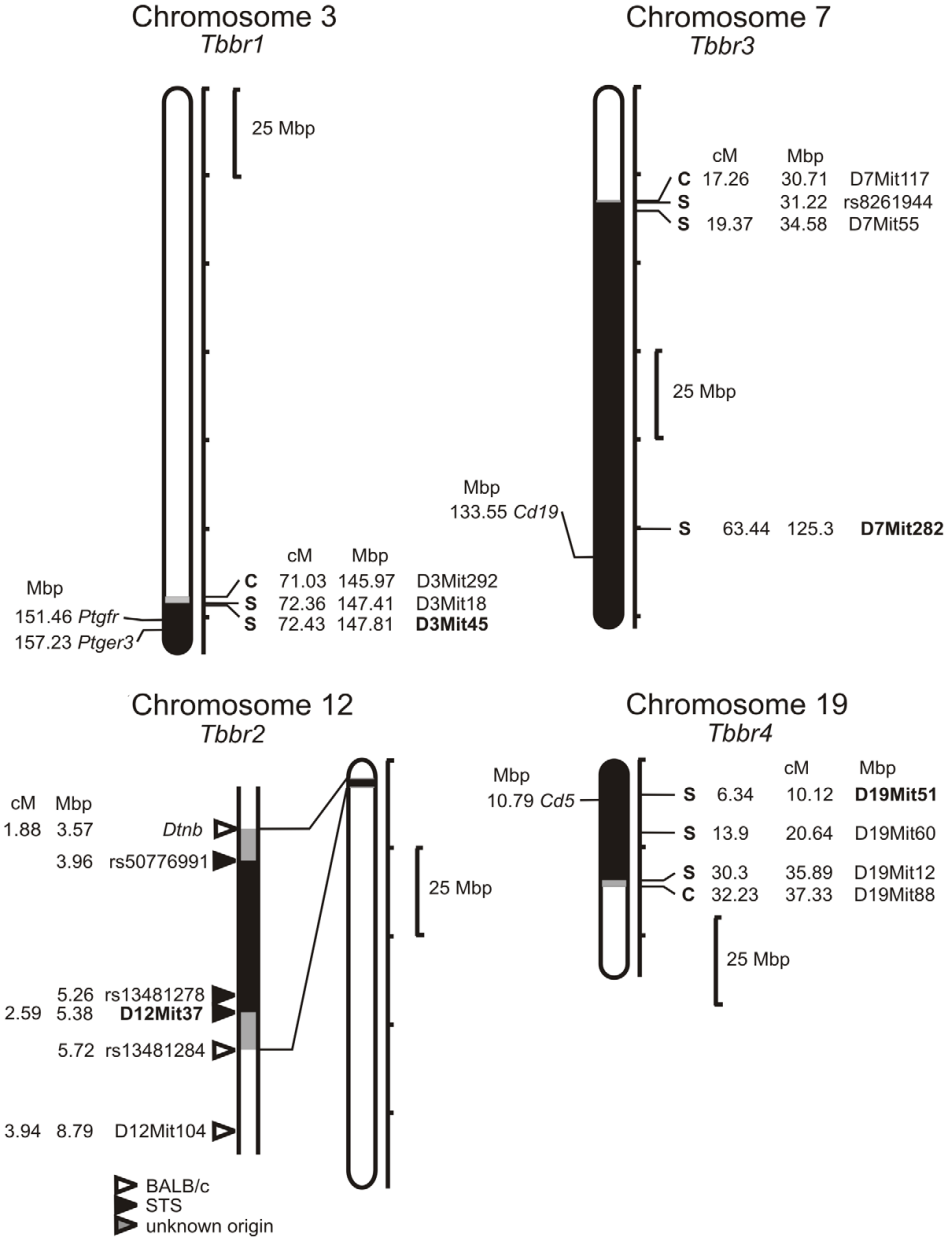
Position of the loci that control response to *T. b. brucei* in strain CcS-11. The regions of STS and BALB/c origin are represented as dark and white, respectively; the boundary regions of undetermined origin are shaded. Only the markers defining the boundaries the STS-derived segment and the markers that were tested for linkage are shown. The markers that exhibit significant *P* values (corrected for genome-wide search) are shown in bold.

*Tbbr2* maps in CcS-11 to a rather short STS-derived region on proximal part of chromosome 12, with previously estimated length of 6 cM [32], [35]. In order to map this locus more precisely, we genotyped this region with 8 microsatellite markers and 4 SNPs. This led to precision mapping of *Tbbr2* to a region with a maximal length of 2.15 Mb that contains only 26 genes (Figure 5).

## Discussion

### CcS-11 differs in susceptibility to trypanosomiasis from both parental strains

CcS-11 differs in susceptibility to trypanosomiasis from both parental strains. The background strain BALB/c is susceptible to *T. b. brucei*. This is in agreement with findings of other research groups [15], [16]. Donor strain STS does not differ in survival from the background strain BALB/c, however the strain CcS-11 that contains a set of approximately 12.5% genes of the donor strain STS and 87.5% genes of the background strain BALB/c and it has shorter survival after infection than either parent. The elements in the BALB/c genome that work in interaction with STS disease response loci can be identified in linkage tests as gene-gene interactions. For example, in the interaction of *Tbbr3* and *Tbbr4*, the survival of mice with homozygous BALB/c alleles at both loci, or homozygous STS alleles at both loci is longer than of mice that are homozygous for BALB/c allele at one locus and homozygous for the STS allele at the second (Table 2A). The fine mapping and molecular identification of *Tbbr4* will reveal one of BALB/c elements that can modify the effect of STS genes. The RC strains are especially suitable to detect such interactions [36].

The observations of progeny having a phenotype, which is beyond the range of the phenotype of its parents are not rare in traits controlled by multiple genes. Some F_2_ hybrids derived in cross between trypanotolerant African N'Dama (*Bos taurus*) and trypanosusceptible Kenya Boran (*Bos indicus*) cattle differed from both parents and contained less *T. congolense* parasites than any of them [37]. Similarly, mouse RC strain OcB-9 differs from both parental strains O20 and B10.O20 in response to alloantigens [38], several RC strains exhibit in some parameters higher susceptibility to *Leishmania major* than both parental strains BALB/c and STS [39], and analysis of gene expression from livers in chromosome substitution strains (background strain C57BL/6, donor strain A/J) revealed that only 438 out of 4209 expression QTLs were inside the parental range [40]. These observations are due to multiple gene-gene interactions of QTLs, which in new combinations of these genes in RC strains, F_2_ hybrids or in chromosomal substitution strains can lead to appearance of new phenotypes that exceed their range in parental strains. Also, with traits controlled by multiple loci, the parental strains often contain susceptible alleles at some of them and resistant on others, and some progeny may receive predominantly susceptible alleles from both parents.

We have compared in strains BALB/c, STS and CcS-11 splenomegaly, hepatomegaly, changes of body weight (Figure 2), and cytokine and chemokine levels (Figure 3). However, none of these measurements explains differences in survival between BALB/c and CcS-11. BALB/c and CcS-11 also do not differ in parasitemia day 10 p.i. (data not shown). Thus, the identification of *Tbbr1*-*Tbbr4* genes is needed to provide information about the mechanisms controlling differences in survival between these strains.

### Susceptibility loci and potential candidate genes

We have detected four loci that in the strain CcS-11 control survival after *T. b. brucei* infection and mapped them with a precision of 1 cM–25 cM (Tables 1, 2, Figure 5). Usually, a standard inbred-strain mapping experiment using F_2_ hybrids will map a QTL onto a 20- to 40-cM interval [41]. Using advanced intercross lines [20], [22] the susceptibility loci *Tir1* and *Tir3c* to *T. congolense* were mapped with a 95% confidence interval to 1.3 and 2.2 cM, respectively. In the RC strains the donor-derived segments of medium length (5–25 cM) comprise 54% of donor genome [42]. However, RC strains can carry on some chromosomes very short segments of donor strain origin. This feature of the RCS system allowed us previously to narrow the location of *Lmr9* (*Leishmania major* response 9) on chromosome 4 to a short segment of 1.9 cM without any additional crosses [43]. The short length of this segment, which controls levels of serum IgE in *L. major* infected mice, enabled us to map a human homolog of this locus on human chromosome 8 and show that it controls susceptibility to atopy [44].

Our data show sex influence on survival as after correction for the genome-wide testing significance of the *Tbbr* loci was detected only in females or in the whole tested group. This observation can be related to the influential role of sex hormones in control of parasitic infections by their ability to modulate different components of both the innate and adaptive immune responses [45], [46]. Greenblatt and Rosenstreich [47] analyzed resistance of the 10 inbred mouse strains and two sets of F_1_ hybrids to infection with *T. b. rhodesiense.* C3H/HeN, C3H/HeJ, CBA/J, BALB/c and CBA/CaJ were highly susceptible, with mean survival times of less than 22 days, and did not exhibit differences in survival between males and females, whereas in more resistant strains CBA/N, A.CA, C57BL/6J, C57BL/KsJ, C57BL/10SnJ, (BALB/c x C57BL/6)F_1_ and (C57BL/6× BALB/c)F_1_ female mice were more resistant than males. These data support the finding of different genetic regulation of susceptibility to *T. brucei* in males and females in certain genetic combinations. Genes controlling infections that appear to be sex dependent have been observed also with other pathogens. For example, *Rmp4* (resistance to mouse pox 4) controls susceptibility to ectromelia virus in female mice only [48] and *Hrl* (herpes resistance locus) exhibits higher influence on susceptibility to Herpes simplex virus in male than in female mice [49]. Sex specific QTLs influence also susceptibility to Theiler's murine encephalomyelitis virus-induced demyelination: loci *Tmved7* and *8* affect male mice only, whereas locus *Tmved9* controls susceptibility only in females. Locus *Tmved6* operates both in females and males, but it has an opposite effect on disease susceptibility in males and females [50]. *Lmr20* influenced IgE level in *L. major* infected females, but not in males [35]. QTLs *Cnes1* and *Cnes2* were associated with high pulmonary *Cryptococcus neoformans* burden in females, whereas *Cnes3* was associated with fungal pulmonary burden in male mice [51]. QTL on chromosome 17 controls susceptibility to pulmonary infection with *Chlamydia pneumoniae*, but has much stronger effect in males, whereas QTL on chromosome 5 controls susceptibility only in female mice [52]. In humans, for example the *IL9* genetic polymorphism (rs2069885) has an opposite effect on the risk of severe respiratory syncytial virus bronchiolitis in boys and girls [53].

In the present study, we were able to precision map *Tbbr2* to 2.15 Mb. This segment contains 26 genes, 12 of them are either predicted genes or cDNA sequences (Table 3). Public databases (http://www.ncbi.nlm.nih.gov; http://www.informatics.jax.org and http://biogps.gnf.org/#goto=welcome) show that some of these genes are in non-infected mice expressed in tissues such as liver, spleen, and brain (Table 3). These organs are in infected mice affected by parasite [6], [7]. There is no obvious candidate gene and there are only indirect indications about the possible role of some of these genes, such as *Dnmt3a* (DNA methyltransferase 3a)[54],[55], *Pomc* (pro-opiomelanocortin-alpha) [56], [57], *Adcy3* (adenylate cyclase 3) [58], and *Ncoa1* (nuclear receptor coactivator 1) [59] in immune response against *Trypanosoma*.

**Table 3:** Expression of genes in locus *Tbbr2* in liver, spleen and brain of non-infected animals.

Gene	ID	Liver		Spleen		Brain	
		NCBI,MGI	BioGPS	NCBI,MGI	BioGPS	NCBI,MGI	BioGPS
*Gm11061, predicted gene 11061*	MGI:3779285	NT	NT	NT	NT	NT	NT
*Dnmt3a, DNA methyltransferase 3A*	MGI:1261827	**YES**	<<M	**YES**	**>M**	**YES**	**>M**
*Gm10485, predicted gene 10485*	MGI:3641689	NT	NT	NT	NT	NT	NT
*Pomc, pro-opiomelanocortin-alpha*	MGI:97742	NO	<<	NO	<<	**YES**	<<
*Efr3b, EFR3 homolog B (S. Cerevisiae)*	MGI:2444851	NO	**>M**	NO	**>M**	**YES**	**>M**
*Dnajc27, Dnaj (Hsp40) homolog, subfamily C, member 27*	MGI:2443036	NO	<M	NO	<M	**YES**	**>30xM**
*Adcy3, adenylate cyclase 3*	MGI:99675	**YES**	<M	**YES**	**>M**	**YES**	**>3M**
*Cenpo, centromere protein O*	MGI:1923800	**YES**	<M	NO	<M	**YES**	<M
*2410017P09Rik, RIKEN cDNA 2410017P09 gene*	MGI:1916959	**YES**	**>M**	NO	<M	**YES**	**>M**
*Ncoa1, nuclear receptor coactivator 1*	MGI:1276523	**YES**	<M	**YES**	<M	**YES**	**>3xM**
*Gm3613, predicted gene 3613*	MGI:3781789	NO	NT	NO	NT	NO	NT
*Gm3620, predicted gene 3620*	MGI:3781796	NO	**>M**	NO	**>M**	NO	**>M**
*Gm3625, predicted gene 3625*	MGI:3781801	NO	NT	**YES**	NT	**YES**	NT
*Itsn2, intersectin 2*	MGI:1338049	**YES**	<M	**YES**	**>3xM**	**YES**	**>M**
*4930417G10Rik, RIKEN cDNA 4930417G10 gene*	MGI:1922105	NO	**>M**	NO	<M	**YES**	**>M**
*A830093I24Rik, RIKEN cDNA A830093I24 gene*	MGI:2442121	**YES**	>M*	NO	>M*	**YES**	>M*
*Pfn4, profilin family member 4*	MGI:1920121	NO	<M**	NO	NT	**YES**	<M**
*Gm6682, predicted gene 6682*	MGI:3647156	NT	**>M**	NT	<M	NT	<M
*0610009D07Rik, RIKEN cDNA 0610009D07 gene*	MGI:1913305	**YES**	<M	**YES**	**>M**	**YES**	<M
*Fkbp1b, FK506 binding protein 1b*	MGI:1336205	NO	<M	NO	<M	**YES**	<M
*BC068281, cDNA sequence BC068281*	MGI:3040699	**YES**	**>3xM**	**YES**	**>M**	**YES**	**>M**
*Mfsd2b, major facilitator superfamily domain containing 2B*	MGI:3583946	NO	<M	**YES**	<M	NO	<M
*Ubxn2a, UBX domain protein 2A*	MGI:2442310	**YES**	**>M**	**YES**	**>M**	**YES**	**>3M**
*Atad2b, ATPase family, AAA domain containing 2B*	MGI:2444798	**YES**	<M	**YES**	**>M**	**YES**	**>M**
*Klhl29, kelch-like 29 (Drosophila)*	MGI:2683857	NO	<M	NO	**>3M**	**YES**	**>30xM**
*2810032G03Rik, RIKEN cDNA 2810032G03 gene*	MGI:1919919	NO	**>M**	NO	<M	**YES**	**>3xM**

Data were compiled from public databases (Http://www.ncbi.nlm.nih.gov; http://www.informatics.jax.org) February 25, 2011 and http://biogps.gnf.org/#goto=welcome, February 25, 2011). NCBI/MGD: YES – expression of a gene was observed; NO – expression of a gene was not observed; NT – not tested. BioGPS: Majority of data were obtained using Gene Atlas MOE430, *Gene Atlas GNF1M, **Gene Atlas U133A. M = median value across all samples for a single probe set. NT – not tested.

*Tbbr1* is localized in the distal part of chromosome 3. Potential candidate genes in this locus are *Ptgfr* (prostaglandin F receptor) [MGI:97796] and *Ptger3* (prostaglandin E receptor 3 (subtype EP3)) [MGI:97795], as prostaglandins play a suppressive role in infection with African trypanosomes [60].

*Tbbr3* on chromosome 7 and *Tbbr4* on chromosome 19 map near to the genes *Cd19* [MGI:88319] and *Cd5* [MGI:88340], respectively, that code markers of B lymphocytes. CD19 is a B-lineage antigen, present on both B-1 and B-2 cells [61]. It was shown that in murine experimental *T. brucei* trypanosomiasis, B-cells were crucial for periodic peak parasitemia clearance and survival of host [16]. CD5^+^ subpopulation of B-1 cell has been found to be stimulated by different *Trypanosoma* species: *T. cruzi* [62], *T. b. evansi* [63], and *T. congolense* [64]. These B-cells were the main source of antibodies reactive with non-parasite antigens in *T. congolense*-infected cattle [64].

However, genes that are presently not considered as possible candidates might cause the effects of some or all *Tbbr* loci. Moreover, not only genes, but also noncoding RNAs in *Tbbr* loci region may influence the outcome of infection [65].

### Are *Tbbr* loci involved in control of other pathogens?

Some genes, for example *Slc11a1* (solute carrier family 11 (proton-coupled divalent metal ion transporters), member 1) or *Lyst* (lysosomal trafficking regulator) */beige* have been found to control susceptibility to several pathogens (reviewed in ref [29]). *Tbbr2* might be potentially involved also in control of *Leishmania major*, as it overlaps with locus *Lmr22 (Leishmania major* response 22*),* which in interaction with *Lmr5* controls serum IL-4 in *L. major* infected mice [35], whereas *Tbbr3* on chromosome 7 maps near to *Ity7* (immunity to *S. typhimurium 7*) [66].

Control of susceptibility to *T. congolense* is exercised by loci on chromosomes 17, 5 and 1 [19], [22], whereas susceptibility to *T. cruzi* is determined by loci on chromosomes 17 and 5 [23]. Influence of loci on chromosomes 17 and 5 could not be tested in the present cross, as CcS-11 does not carry STS-derived segments on these chromosomes [32]. STS-derived region present on chromosome 1 of CcS-11 overlaps with *Tir3c* [22], however we did not detect influence of this segment on susceptibility to *T. b. brucei.* This might be caused either by differences in regulation of immunity against the sub-genus *T. (Nannomonas) congolense* and the subgenus *T. (Trypanozoon) brucei,* or because the *Tir3c,* which was detected in a cross between strains C57BL/6J and BALB/c [19] and C57BL/6J and A/J [22] is not polymorphic between strains BALB/c and STS tested in this paper. Therefore the possible effects of *Tbbr* loci in infection with other *Trypanosoma* species have yet to be established.

In summary, this study represents the first definition of genetic loci controlling susceptibility to *T. b. brucei* infection. One of them, *Tbbr2* is precisely mapped to the segment that contains only 26 genes, which will facilitate the identification of the candidate gene.

*T. brucei* subspecies cause sleeping sickness in humans and affect also all livestock, with particularly severe effects in horses and dogs [1]. Thus, the definition of genes controlling anti-parasite responses might also permit a better understanding of pathways and genetic diversity underlying the disease phenotypes in humans and domestic animals.

## Supporting Information

Figure S1 **Differences in levels of CCL4/MIP-1β, CCL5/RANTES, and TNF-α between infected and non-infected mice.** Female mice strains of BALB/c (11 infected tested 2^nd^ day p.i., 22 infected tested 10^th^ day p.i., 22 non-infected), STS (9 infected tested 2^nd^ day p.i., 17 infected tested 10^th^ day, 13 non-infected) and CcS-11 (14 infected tested 2^nd^ day p.i., 25 infected tested 10^th^ day p.i., 26 non-infected) were compared. Animals were intra-peritoneally inoculated with 2.5×10^4^ bloodstream forms of *T. b. brucei.* Control, non-infected mice were kept in the same animal facility. Mice were killed 10 days after inoculation. The data show the means ± SD from three independent experiments.
(TIF)

Table S1 **Survival times and genotypes of F_2_ hybrids between BALB/c and CcS-11.**
(XLS)

Table S2 ***P*** **values of differences in serum chemokines and cytokines levels between non-infected and infected mice.**
(DOC)
