# Supplementary figures and images for: Genetic Control of Resistance to Trypanosoma brucei brucei Infection in Mice
Source: PLoS Negl Trop Dis. 2011 Jun 7;5(6):e1173. doi: 10.1371/journal.pntd.0001173 (PMC3110168; doi:10.1371/journal.pntd.0001173)

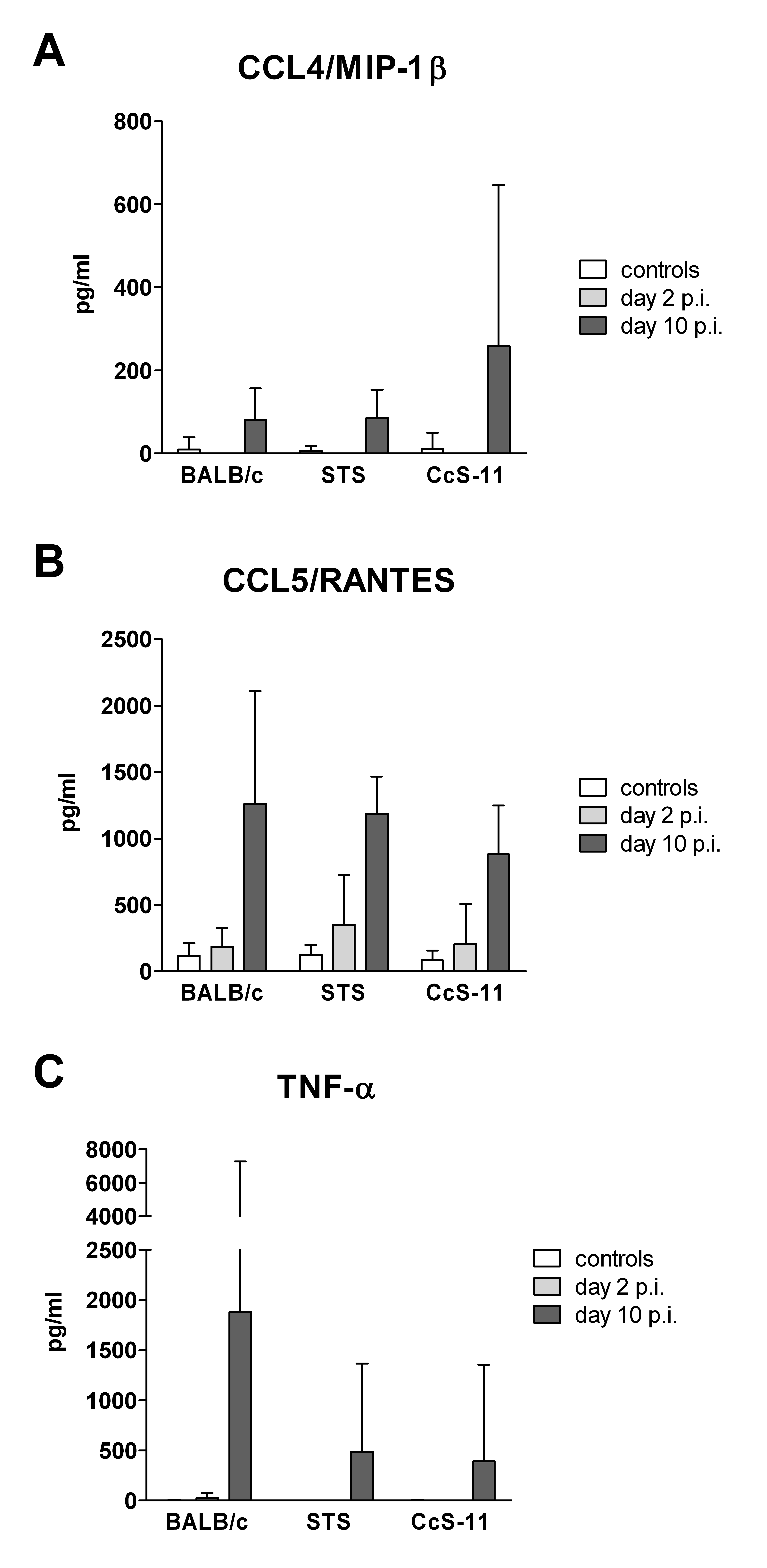

Supplement: Figure S1 — Differences in levels of CCL4/MIP-1β, CCL5/RANTES, and TNF-α between infected and non-infected mice. Female mice strains of BALB/c (11 infected tested 2nd day p.i., 22 infected tested 10th day p.i., 22 non-infected), STS (9 infected tested 2nd day p.i., 17 infected tested 10th day, 13 non-infected) and CcS-11 (14 infected tested 2nd day p.i., 25 infected tested 10th day p.i., 26 non-infected) were compared. Animals were intra-peritoneally inoculated with 2.5×10^4^ bloodstream forms of T. b. brucei. Control, non-infected mice were kept in the same animal facility. Mice were killed 10 days after inoculation. The data show the means ± SD from three independent experiments. (TIF) [file pntd.0001173.s001.tif]
