# Supplementary material for: Genetic Control of Resistance to Trypanosoma brucei brucei Infection in Mice
Source: PLoS Negl Trop Dis. 2011 Jun 7;5(6):e1173. doi: 10.1371/journal.pntd.0001173 (PMC3110168; doi:10.1371/journal.pntd.0001173)
Supplement: Table S2 — P values of differences in serum chemokines and cytokines levels between non-infected and infected mice. (DOC) [file pntd.0001173.s003.doc]

**Table S2. *P* values of differences in serum chemokines and cytokines l**evels between non-infected and infected mice.

| **Strain** | **day p.i.** | **CCL2/MCP-1** | **CCL3/MIP-1α** | **CCL4/MIP-1β** | **CCL5/RANTES** | **CCL7/MCP-3** | **TNF-α** | **GM-CSF** |
| --- | --- | --- | --- | --- | --- | --- | --- | --- |
| **BALB/c** | 2 | NS | NS | NS | NS | 0.00017 | NS | NS |
|  | 10 | 0.0000001 | 0.000067 | 0.00024 | 0.000001 | 0.0000001 | 0.000001 | NS |
| **STS** | 2 | NS | NS | NS | 0.005 | 0.045 | NS | NS |
|  | 10 | 0.000012 | 0.000003 | 0.00058 | 0.000004 | 0.000004 | 0.000002 | NS |
| **CcS-11** | 2 | NS | NS | NS | NS | 0.001 | NS | NS |
|  | 10 | 0.0000001 | 0.000004 | 0.000001 | 0.0000001 | 0.0036 | 0.000001 | NS |
